# Inequalities in cancer 2-week-wait referrals: a cross-sectional study in English general practice

**DOI:** 10.3399/BJGPO.2024.0052

**Published:** 2025-06-18

**Authors:** Stephanie C Wynne, Mark Ashworth

**Affiliations:** 1 Rehabilitation and Therapies Department, Guy’s and St Thomas’ NHS Foundation Trust, London, UK; 2 School of Life Course and Population Sciences, Faculty of Life Sciences and Medicine, King’s College London, London, UK

**Keywords:** Cancer, Early diagnosis, Primary care

## Abstract

**Background:**

Practices with higher 2-week-wait (2WW) referral rates demonstrate higher survival for several cancers. Yet, there is little up-to-date evidence exploring factors influencing 2WW referral rates and whether health inequalities exist, particularly after COVID-19.

**Aim:**

To establish which patient factors (for example, age, sex, ethnic group, deprivation) and practice factors (for example, remote consultations, frequency of seeing a preferred GP) independently predict 2WW referral rates.

**Design & setting:**

A cross-sectional, observational study was performed using data from English general practices for 2021–2022.

**Method:**

Multivariable linear regression was used to identify the strongest, independent predictors of 2WW referral rates for all cancers (primary outcome) and for breast, lower-gastrointestinal, lung, and skin cancers separately (secondary outcome).

**Results:**

The analysis included 6307 practices. Practices with more females, patients aged ≥75 years, and patients with a greater burden of long-term conditions were associated with higher 2WW referrals for all cancers, as were practices in Northwest England, and those with higher scores for patients feeling involved in care decisions. Conversely, practices with a higher frequency of seeing a preferred GP were predictive of fewer all-cancer 2WW referrals. Practices with a higher proportion of currently smoking patients and Asian and Black ethnicity patients also predicted fewer all-cancer 2WW-referrals, and these associations were strongest for skin cancer, and for breast cancer (except for Black ethnicity). Higher socioeconomic deprivation predicted lower 2WW referrals for lung cancer only.

**Conclusion:**

This study analyses factors influencing 2WW referral rates and highlights potential inequalities. This work identifies priority populations, including people who smoke, and Asian and Black ethnic group patients, who may benefit from interventions to increase primary care access. Shared decision making may be an underexplored resource for increasing all-cancer 2WW referral rates.

## How this fits in

Inequalities in cancer staging and survival exist in the UK. The COVID-19 pandemic exacerbated many existing health inequalities and impacted primary care delivery, thus an updated understanding of avoidable disparities in 2WW activity is merited. Using data from 2021–2022, when 2WW referral rates had returned to pre-pandemic levels, this study examines patient and practice-level factors that influence 2WW activity. It provides recommendations to improve equitable use of 2WW pathways in future.

## Introduction

Early cancer diagnosis is essential for improving survival and access to curative treatment. Currently, only 52% of cancers in the UK are diagnosed at stage I–II,^1,2^ with a target of 75% by 2028.^1^ Despite this, inequalities exist in cancer outcomes in the UK. Asian and Black ethnic groups and patients living in more socioeconomically deprived neighbourhoods experience higher odds of late-stage cancer diagnoses compared with White individuals residing in less deprived communities.^3–5^ Thus, it is necessary to identify inequalities in cancer care, including 2WW pathways, to reduce this disparity.

Also termed the ‘faster diagnosis standard’, 2WW pathways aim to expedite cancer diagnoses by requiring GPs to refer patients with potential cancer symptoms for specialist investigation within 2 weeks.^6^ Practices sending higher 2WW referrals are associated with improved cancer outcomes (fewer stage III–IV diagnoses and lower mortality rates) for several cancers.^2,7^ Consequently, the National Institute for Health and Care Excellence updated the 2WW guidelines in 2015 to encourage greater use of this pathway by reducing the risk threshold for referral from 5% to 3%.^6^

Several studies have identified factors associated with higher 2WW referrals including: older age,^8–11^ female sex,^8,9,12^ White ethnic group,^8,11,13^ lower GP–patient continuity of care,^14,15^ and practices with registrars in primary care specialty training.^8,16^ However, most studies precede 2015 changes to 2WW guidelines and pre-date the impact of COVID-19.^17,18^ Hence, this study aimed to update the existing evidence base by identifying the strongest, independent predictors of 2WW referrals for all cancers during 2021–2022, and then for breast, lower-gastrointestinal, lung and skin cancers separately.

## Method

### Design

This retrospective cross-sectional study analysed practice-level data from 2021 to 2022. English general practices with a list size <500 (1.3%) were excluded as atypical^19^ and 1.2% were removed for missing 2WW data, leaving *n* = 6307 practices remaining.

The primary outcome, number of all-cancer 2WW referrals per 100 000 population, was presented as a rate (number of 2WW referrals × 100 000/list size). Predictor variables (Tables 1 and 2) were selected based on a comprehensive literature review^8–16^ and to explore pertinent gaps (for example, the relationship between remote consultations and 2WW referrals).^20^

Data on 2WW referrals was derived from the Office for Health Improvement and Disparities dashboards.^21^ Descriptive data on population characteristics (for example, age and sex), practice characteristics (for example, list size), and payments to general practice (for example, extended-hours access) were sourced from NHS Digital.^22^ Data on ethnic group and deprivation were derived from the 2011 National Census^23^ and 2019 Index of Multiple Deprivation (IMD),^24^ respectively. Deprivation and ethnic group variables were patient attributed, meaning scores were adjusted for the lower super output area of residence of the practice-registered population and not on the location of the general practice.^25^

Several measures of patient experience were sourced from the General Practice Patient Survey (GPPS).^26^ The Quality and Outcomes Framework (QOF) provided data on quality of clinical care (QOF total score),^27^ rate of people who currently smoke, and enabled the calculation of a composite ‘morbidity index’. QOF indicator ‘SMOK002’ records the number of patients with at ≥1 of a subset of nine long-term conditions (LTCs);^28^ see Table 1. The denominator of this QOF indicator was used to calculate a ‘morbidity index’ (rate of LTCs per 1000 registered patients) for each practice.

**Table 1.** Descriptive statistics for patient-related factors for all practices submitting 2WW referrals per 100 000 population for all cancers between 2021 and 2022 (*n* = 6307)

Primary outcome	Mean	SD	10th Centile	90th Centile
**2WW referral rate for all suspected cancers** (**number per 100 000 population per year**)	**4307.3**	**1536.6**	**2442.6**	**6260.6**
**Predictor variables**				
Cancer emergency presentations (number per 100 000 population per year)	90.0	47.8	32.3	149.4
Female, %	49.7	2.3	47.1	51.6
Age <30 years, % Age 30–74 years, % Age ≥75 years, %	34.9 56.7 8.4	6.8 4.6 3.8	28.1 52.9 3.3	42.5 60.4 13.4
White, % Mixed/multiple ethnic groups, % Asian/Asian British, % Black/African/Caribbean, % Other ethnic group, %	79.0 3.0 10.9 4.7 2.4	21.3 1.9 14.4 6.4 2.9	45.2 1.0 0.9 0.3 0.3	97.4 6.0 29.6 12.7 6.3
**GPPS Q58 – Religion**				
No Religion, % Buddhist, % Christian, % Hindu, % Jewish, % Muslim, % Sikh, % Other, %	28.3 0.7 56.6 2.2 0.6 5.6 1.0 1.5	9.8 1.1 13.4 5.7 3.4 12.1 3.9 1.3	15.1 0.0 39.2 0.0 0.0 0.0 0.0 0.0	39.2 1.9 70.2 5.2 1.0 15.5 2.1 3.2
Morbidity index^a^ (rate per 1000 registered patients)	233.4	5.2	164.4	293.4
Depression, QOF prevalence, %	12.6	4.5	7.2	18.5
Learning disabilities, QOF prevalence, %	0.6	0.3	0.3	0.9
People who currently smoke (aged ≥15 years) (rate per 1000 registered patients)	128.0	41.8	78.0	180.9
Patient-attributed IMD score	23.3	11.6	10.1	39.7
**GPPS Q50 – employment status**				
Employed full time, % Employed part time, % Full-time education, college or university, % Unemployed, % Permanently sick/disabled, % Fully retired, % Looking after the family or home, % Doing something else, %	34.2 12.6 2.2 3.6 4.7 35.0 4.8 2.9	8.0 3.6 3.5 3.4 3.2 11.4 2.8 1.9	25.0 8.0 0.0 0.8 1.1 18.4 1.8 0.9	44.4 17.1 4.3 7.8 9.0 48.2 8.2 5.3
GPPS Q56 - Responder has family caring responsibilities, % yes	20.5	4.7	14.6	26.4

GPPS = Ipsos MORI General Practice Patient Survey. IMD = Indices of Multiple Deprivation 2019. QOF = Quality and Outcomes Framework. SD = standard deviation. 2WW = 2-week-wait referral for cancer.

^a^Morbidity index calculated as the rate of practice-registered patients with ≥1 of nine predefined QOF long-term conditions: coronary heart disease, peripheral artery disease, stroke/transient ischaemic attack, hypertension, diabetes, chronic obstructive pulmonary disease, chronic kidney disease, asthma, and schizophrenia/bipolar affective disorder.

### Statistical analysis

A detailed flow diagram of the statistical methods are outlined in Supplementary File S1. Descriptive statistics were provided as mean, standard deviation (SD), and 10th to 90th percentile (continuous variables) or frequency and percentage (categorical variables).

A Pearson correlation or independent-samples *t*-test assessed univariable relationships between 2WW referral rate and each continuous or binary predictor variable, respectively. Predictor variables with a linear relationship and significant at *P*<0.1 on univariable analysis were then entered into a multivariable linear regression (MVR) model simultaneously.^29^ Prior to analysis, the assumptions of MVR were tested, and multicollinearity was assessed, considering a variance inflation factor (VIF) <10 acceptable.^30^ Reference categories (see Table 3a, b footnote) were selected as the lowest relevance group when considering 2WW referral activity (for example, age <30 years^9^).

MVR was performed to detect independent associations between 2WW referrals and each predictor variable, rank the strongest predictor variables, and control for confounding factors (variables that may compete with another predictor variable when explaining any observed changes in the outcome variable).^31^ Three outputs included: (1) an adjusted *R* ^2^ value indicating the percentage of variance in 2WW referrals explained by all predictor variables; (2) a standardised beta coefficient ranking the strength of association between 2WW referrals and each predictor variable; and (3) an unstandardised B-coefficient (95% confidence interval [95% CI]) demonstrating the change in 2WW referrals per unit change in each predictor variable.

To simplify the interpretation of the MVR analysis, a stepwise linear regression function was then performed to produce a final, parsimonious model demonstrating the highest adjusted *R*² value that could be attained from the five strongest predictors of 2WW referrals at *P*<0.05 significance.

Sensitivity analyses were repeated, as above, for the four separate cancer sites. All analyses were completed using IBM SPSS Statistics (version 28.0).

## Results

### Descriptive data

The mean (10th to 90th centile) number of 2WW referrals per 100 000 population for all cancers in 2021–2022 was 4307.3 (2442.6–6260.6), equating to around 4% of the population receiving a 2WW referral.

For patient-related variables (Table 1), a third of practice-registered patients were aged <30 years with a smaller proportion (mean 8.4%; SD 3.8%) aged ≥75 years. The majority (mean 79.0%; SD 21.3%) were of White ethnic group with a mean IMD score of 23.3 (SD 11.6), where a larger IMD score reflects higher deprivation.^24^ The mean morbidity index was 233.4 (SD 5.2) per 1000 practice-registered patients, demonstrating that just under a quarter of patients had at least one LTC. The mean rate of currently smoking patients per 1000 practice-registered patients was 128.0 (SD 41.8).

For practice-related variables (Table 2), the mean practice list size (number of patients per GP full-time equivalent) was 2242.8 (SD 1735.8) and almost half of practices were training practices. QOF total score showed only 120 practices (1.9%) not achieving 100% of the available points. However, a COVID-19 payment-protection scheme may have inflated these scores.^27^ There were slightly more remote consultations (mean 51.1%; SD 12.9%) than in-person ones (mean 48.9%; SD 12.9%). Frequency of seeing a preferred GP was generally low (mean 39.5%; SD 17.6%), with marked variation across practices (10th to 90th centile: 18.2–63.6%).

**Table 2.** Descriptive statistics for general practice-related factors for all practices submitting 2WW referrals per 100 000 population for all cancers between 2021 and 2022 (*n* = 6307)

Characteristic	Mean	SD	10th Centile	90th Centile
**Practice staffing (FTE per 1000 registered patients) and QOF achievement**				
Practice list size (number of registered patients per GP FTE)	2242.8	1735.8	1080.0	3551.0
Permanent qualified GPs	0.4	0.2	0.2	0.7
Locum GPs	0.02	0.05	0.00	0.06
Total nurses	0.3	0.2	0.1	0.4
Total receptionists	0.5	0.3	0.2	0.8
Total QOF score^a^ – maximum points achieved (%)	100.0	0.5	100.0	100.0
**Practice location and characteristics**	%			
Midlands	20.1			
London	18.1			
Northwest England	15.2			
North East and Yorkshire	15.1			
Southeast England	12.8			
East of England	10.1			
Southwest England	8.5			
Urban versus rural (% urban)	83.5			
GP extended-hours access (% yes)	5.2			
Training practice (% yes)	48.9			
**GPPS respondent scores^b^ (for the last appointment unless stated otherwise)**	**Mean**	**SD**	**10th Centile**	**90th Centile**
Q13 – Remote telephone/video/online (%)	51.1	12.9	33.3	66.7
Q13 – In-person/in practice/at home (%)	48.9	12.9	33.3	66.7
Q3 – Ease of getting through to the practice via phone (% rating ‘very–fairly easy’)	58.8	22.3	27.4	88.4
Q114 – Used GP online services to book an appointment (% yes in the past 12 months)	17.7	10.7	6.9	32.5
Q25 – Satisfaction with GP appointment times (% rating ‘very–fairly satisfied’)	59.6	14.0	41.2	77.9
Q18 – Overall experience of making an appointment (% rating ‘very–fairly good’)	60.5	15.7	39.6	81.0
Q111 – Patient avoided making a GP appointment (% yes in the past 12 months)	51.2	9.4	39.1	63.5
Q9 – Frequency of seeing a preferred GP (% rating ‘always’ and ‘a lot’)	39.5	17.6	18.2	63.6
Q86a – HCP gave enough time (% rating ‘very-fairly good’)	85.3	7.5	75.0	93.9
Q86b – Felt listened to by HCP (% rating ‘very-fairly good’)	86.2	7.0	76.7	94.3
Q90 – Felt needs were met (% rating ‘definitely–to some extent’)	92.6	4.4	86.7	97.5
Q88 – Felt involved in decisions about treatment and care (% rating ‘very–fairly good’)	91.1	5.3	83.8	97.1
Q86e – Was treated with care and concern (% rating ‘very–fairly good’)	85.4	7.4	75.3	94.0
Q89 – Had confidence and trust in the HCP (% rating ‘definitely–to some extent’)	94.1	4.1	88.7	98.7

FTE = full-time equivalent. GPPS = Ipsos MORI General Practice Patient Survey. HCP = healthcare professional. QOF = Quality and Outcomes Framework. SD = standard deviation. 2WW =2-week-wait referral for cancer.

a Participation in QOF is voluntary, although rates are high (97.5% coverage during 2021-2022).^32^

b Mean (SD) GPPS response rate for the included practices: 32.2% (10.0%).

### MVR (all cancer 2WW referral rate)

Results from the univariable analysis are provided in Supplementary Tables S2 and S3. There were significant associations between all-cancer 2WW referrals and all predictor variables, except for learning disability prevalence, two locations (Southeast England; Northeast and Yorkshire), and two GPPS variables (Q3: ease of reaching the practice via phone; Q25: satisfaction with appointment times).

All significant variables were entered into a MVR model except for QOF total score (highly skewed) and two GPPS variables (Q50: fully retired; Q86b: felt listened to by the healthcare professional) because of multicollinearity (VIF >10).

The MVR model for all-cancer 2WW referrals containing all eligible predictor variables is provided in Table 3a, b (see Supplementary File S4 for full description). This MVR model had an adjusted *R* ^2^ of 48.8%, indicating that approximately half of the variation in 2WW referral rate could be explained by the included predictors.

**Table 3.** (a) Multivariable linear regression (MVR) model showing significant associations between 2WW referrals per 100 000 population for all cancers and patient and practice-related variables 2021–2022. (b) Multivariable linear regression (MVR) model showing 2WW referrals per 100 000 population for all cancers and patient and practice-related variables 2021–2022 (non-significant variables only)^a^

**a) Significant variables (** ***P*** **<0.05)**						
**Variable (adjusted *R* ^2^ = of 48.8%**)	**ß**	**B** ^b^	**Lower 95% CI for B**	**Upper 95% CI for B**	***P* value**	**VIF**
Age ≥75 years (%)	0.25	101.3	78.9	123.6	<0.001	8.8
Morbidity index per 1000 registered patients	0.21	6.2	4.6	7.8	<0.001	9.3
Practice in London	0.15	609.3	471.5	747.1	<0.001	3.7
Q9 – Frequency of seeing a preferred GP	-0.14	-12.2	-14.6	-9.8	<0.001	2.4
People who currently smoke per 1000 registered patients	-0.14	-5.2	-6.5	-3.8	<0.001	4.1
Practice in Northwest England	0.13	544.8	444.7	644.9	<0.001	1.7
Q18 – Overall experience of making appointment	-0.13	-12.6	-16.4	-8.7	<0.001	4.9
Sex, female (%)	0.09	61.6	43.4	79.8	<0.001	2.2
Depression, QOF prevalence (%)	0.09	32.3	23.6	41.1	<0.001	2.1
Age 30–74 years (%)	0.09	32.3	21.7	42.9	<0.001	2.9
Mixed/multiple ethnic groups (%)	0.09	73.4	40	106.7	<0.001	5.2
Q88 – Felt involved in care decisions	0.07	20.7	9.8	31.7	<0.001	4.6
Q86e – Was treated with care and concern	0.07	14	3.3	24.8	<0.05	8.3
Asian/Asian British (%)	-0.06	-6.6	-11.7	-1.8	<0.05	7
Black/African/Caribbean (%)	-0.06	-14.4	-23.4	-5.5	<0.01	4.4
Permanent qualified GPs (FTE) per 1000 patients	0.05	404	180.9	627.1	<0.001	2
Practice in the East of England	0.05	232.6	127.5	337.6	<0.001	1.3
Training practice (yes)	0.05	147	80.3	213.6	<0.001	1.5
Q90 – Felt needs were met during appointment	0.05	18.6	5.5	31.7	<0.01	4.5
Q58 – Religion: Muslim (%)	-0.05	-6.6	-12.9	-0.3	<0.05	7.7
Q58 – Religion: Sikh (%)	-0.04	-13.9	-23.5	-4.2	<0.01	1.9
Practice in Southwest England	0.04	208.5	95.7	321.3	<0.001	1.3
Q58 – Religion: Other (%)	0.03	37.1	14.4	59.8	<0.01	1.2
Locum GPs (FTE) per 1000 patients	0.03	1003.9	426.7	1581.1	<0.01	1.2
Cancer emergency presentations (per 100 000 population)	-0.03	-1.1	-1.8	-0.3	<0.01	1.5
Q50 – Employment status: unemployed (%)	0.03	15.5	1.9	29	<0.05	2.8
Q50 – Employment status: employed part time (%)	-0.02	-9.8	-18.8	-0.8	<0.05	1.4
Practice in the Midlands	0.02	92.9	8.7	177.2	<0.05	1.5
**b) Non-significant variables (** ***P*** **≥0.05)**						
**Variable (adjusted *R* ^2^ = of 48.8%**)	ß	**B**	**Lower 95% CI for B**	**Upper 95% CI for B**	***P* value**	**VIF**
Q111 – Patient avoided making a GP appointment	-0.03	-4.7	-9.7	0.3	0.06	2.9
Total receptionists (FTE per 1000 patients)	0.02	1.1	0	2.3	0.06	1.2
Total nurses (FTE per 1000 patients)	-0.02	-1.6	-3.7	0.5	0.13	1.4
Registered patients per GP FTE (list size)	0.02	0.02	-0.01	0.04	0.13	1.9
Q58 – Religion: Buddhist	-0.02	-24.2	-52.5	4.1	0.09	1.2
Q58 – Religion: Hindu	-0.02	-5.6	-13.6	2.4	0.17	2.7
Patient-attributed IMD score	0.02	2.4	-3.2	7.9	0.41	5.5
Q58 – Religion: Jewish	-0.01	-6.6	-16.5	3.3	0.19	1.5
Urban versus rural (% urban)	-0.01	-56.3	-146.4	33.8	0.22	1.5
GP extended-hours access (% yes)	0.01	70.8	-63.9	205.5	0.3	1.2
Q50 – Full-time education, college or university (%)	0.01	6.9	-6.4	20.2	0.31	2.4
Q56 - Family caring responsibilities (% yes)	0.01	3.2	-3.5	9.9	0.35	1.3
Q50 – Employment status: permanently sick/disabled (%)	0.01	5.5	-7.3	18.2	0.4	2.2
Q50 - Employed full-time (%)	-0.01	-2.6	-8.9	3.7	0.42	3.3
Q13 – Remote appointment (%)	0	-0.5	-3	1.9	0.69	1.3
Q114 - Used GP online services to book an appointment in the past 12 months (% yes)	0	-0.6	-3.5	2.3	0.69	1.3
Q50 – Doing something else (%)	0	-3.9	-18.4	14.5	0.82	1.3
Q58 – Religion: Christian	0	0.4	-4.3	5.1	0.86	5.2
Other ethnic group (%)	0	-1.4	-20.6	17.7	0.89	4
Q89 – Had confidence and trust in the HCP	0	-1	-15.9	13.9	0.9	4.8
Q86a - HCP gave enough time	0	-0.2	-11	11	0.98	8.7

CI = confidence interval. FTE = full-time equivalent. HCP = healthcare professional. IMD = Indices of Multiple Deprivation 2019. QOF = Quality and Outcomes Framework. SD = standard deviation. 2WW = 2-week-wait referral for cancer. VIF = Variable inflation factor (measure of multicollinearity).

^a^Results are ranked by standardised beta coefficient, where a larger beta denotes a stronger association between 2WW referrals and each predictor variable. Reference variables: Age<30 years (%); White ethnic group (%); General Practice Patient Survey (GPPS) Q50: employment status - Looking after the family or home (%); General Practice Patient Survey (GPPS) Q58: Religion – No religion; General Practice Patient Survey (GPPS) Q13: Remote consultation – in practice or at home (%).Models alternately excluding one of the seven regions as a reference variable were performed. The model with the highest adjusted *R* ^2^ value (using Southeast England as a reference variable) was maintained.

^b^B denotes the increase (+) or decrease (-) in 2WW referrals for every 1% change in continuous predictor variable or changing from a ‘no’ to ‘yes’ status for binary variables.

The parsimonious model (Table 4) identified four strongest positive predictors of all-cancer 2WW referral rate (largest positive beta coefficient): age ≥75 , female sex, patients feeling involved in decisions about care, and practice location in Northwest England. Interpreting the unstandardised B-coefficient (95% CI), a 1% increase in practice-registered patients aged ≥75 years was associated with 160.6 (95% C = 151.4 to 169.9) more all-cancer 2WW referrals per 100 000 population. Conversely, the strongest negative predictor (largest negative beta coefficient) of all-cancer 2WW referrals was a higher frequency of seeing a preferred GP, where a 1% increase in this variable was associated with 17.0 (95% C = 15.2 to 18.8) fewer all-cancer 2WW referrals.

**Table 4.** Parsimonious multivariable linear regression (MVR) model for all-cancer 2WW referrals per 100 000 population for 2021–2022^a^

Variable	ß	B^b^	Lower 95% CI for B	Upper 95% CI for B	*P* value	VIF
**All-cancer 2WW referrals (adjusted *R* ^2^ 43.0%**)						
Age ≥75 years, %	0.40	160.6	151.4	169.9	<0.001	1.5
Q9 – Frequency of seeing a preferred GP^c^	-0.19	-17.0	-18.8	-15.2	<0.001	1.2
Female, %	0.18	123.2	108.3	138.2	<0.001	1.4
Q88 - Felt involved in care decisions about care^d^	0.16	47.1	40.5	53.7	<0.001	1.5
Practice in Northwest England	0.13	562.7	482.6	642.8	<0.001	1.0

CI = confidence interval. 2WW = 2-week-wait referrals for suspected cancer. VIF = variable inflation factor (measure of multicollinearity).

^a^Results are ranked by standardised beta coefficient, where a larger beta denotes a stronger association between 2WW referrals and each predictor variable. ^b^B denotes the increase (+) or decrease (-) in 2WW referrals for every 1% change in continuous predictor variable or changing from a ‘no’ to ‘yes’ status for binary variables. ^c^Percentage of patients rating ‘always’ or ‘a lot’ for frequency of seeing a preferred GP. ^d^Percentage of patients rating their healthcare professional as ‘very good’ or ‘fairly good’ at involving them in decisions about treatment/care.

### Parsimonious models by cancer site (sensitivity analyses)

Sensitivity analyses demonstrated a mean number of 2WW referrals per 100 000 population for suspected breast, lower-gastrointestinal and skin cancer of 817.1 (SD 278.5), 796.2 (SD 351.3) and 886.5 (SD 466.7) respectively. Lung 2WW referrals were substantially lower (86.0, SD 45.1).

MVR models for each cancer site are detailed, in full, in Supplementary Tables S5-S8. However, results of the simplified, parsimonious models are presented in Table 5.

**Table 5.** Parsimonious multivariable linear regression (MVR) models for 2WW referrals per 100 000 population by suspected cancer site (sensitivity analyses) for 2021–2022^a^

Variable	ß	B^b^	Lower 95% CI for B	Upper 95% CI for B	***P* value**	VIF
**Breast 2WW referrals (adjusted *R* ^2^ 29.0%**)						
Female, %	0.28	34.9	31.9	37.9	<0.001	1.4
Asian/Asian British ethnicity, %	-0.28	-5.4	-5.9	-4.9	<0.001	1.6
Age 30–74 years, %	0.16	10.0	8.6	11.4	<0.001	1.2
Morbidity index per 1000 registered patients	0.08	0.4	0.2	0.6	<0.001	3.1
Age ≥75 years, %	-0.08	-6.0	-8.9	-3.1	<0.001	3.6
**Lower GI 2WW referrals (adjusted *R* ^2^ 39.3%**)						
Morbidity index per 1000 registered patients	0.28	1.9	1.7	2.2	<0.001	3.1
Age ≥75 years , %	0.26	24.3	20.9	27.7	<0.001	3.5
Practice in Northwest England	0.19	191.3	171.7	210.9	<0.001	1.1
Q9 – Frequency of seeing a preferred GP^c^	-0.18	-3.5	-4.0	-3.1	<0.001	1.2
Q88 – Felt involved in care decisions about care^d^	0.16	10.6	9.1	12.2	<0.001	1.5
**Lung 2WW referrals (adjusted *R* ^2^ 9.9%**)						
Morbidity index per 1000 registered patients	0.27	0.2	0.2	0.3	<0.001	1.0
People who currently smoke per 1000 registered patients	0.12	0.1	0.1	0.2	<0.001	2.0
Patient-attributed IMD score	-0.12	-0.5	-0.6	-0.3	<0.001	2.1
Q9 – Frequency of seeing a preferred GP^c^	-0.09	-0.6	-0.3	-1.2	<0.001	1.0
Training practice, % yes	0.07	6.4	4.1	8.7	<0.001	1.0
**Skin 2WW referrals (adjusted *R* ^2^ 36.2%**)						
Age ≥75 years, %	0.38	46.4	42.6	50.3	<0.001	2.4
Asian/Asian British ethnic groups, %	-0.28	-9.0	-9.8	-8.2	<0.001	1.6
Mixed/Multiple ethnic groups, %	0.23	57.1	48.8	65.4	<0.001	2.8
Black/African/Caribbean ethnic group, %	-0.18	-13.2	-15.6	-10.9	<0.001	2.6
People who currently smoke per 1000 registered patients	-0.12	-1.4	-1.6	-1.1	<0.001	1.3

CI = confidence interval. GI = gastrointestinal. VIF = variable inflation factor (measure of multicollinearity). 2WW = 2-week-wait referrals for suspected cancer.

^a^Results are ranked by standardised beta coefficient, where a larger beta denotes a stronger association between 2WW referrals and each predictor variable.^b^B denotes the increase (+) or decrease (-) in 2WW referrals for every 1% change in continuous predictor variable or changing from a ‘no’ to ‘yes’ status for binary variables. ^c^Percentage of patients rating ‘always’ or ‘a lot’ for frequency of seeing a preferred GP. ^d^Percentage of patients rating their healthcare professional as ‘very good’ or ‘fairly good’ at involving them in decisions about treatment/care.

Three variables consistently predicted a higher 2WW referral rate across models: female sex (except lung); higher morbidity index (except skin), and practice location in Northwest England (except breast).

Practices with more patients feeling involved in decisions about care were consistently associated with a higher 2WW referral rate, while higher scores for seeing a preferred GP consistently predicted fewer referrals. These associations were particularly strong for suspected lower-gastrointestinal cancer.

Distinct patterns emerged by cancer site. Age ≥75 years was strongly associated with fewer breast 2WW referrals despite predicting higher all-cancer 2WW referrals. Asian ethnic group predicted fewer 2WW referrals for all cancers, as well as for breast and skin cancer, where this association was especially strong. Similarly, Black ethnic group strongly predicted fewer skin 2WW referrals, and to a lesser extent, fewer all-cancer 2WW referrals. Deprivation exhibited a significant association only with lung 2WW referral rate. Finally, current smoking status predicted a higher lung 2WW referral rate but a lower 2WW referral rate for all cancers and for skin cancer.

## Discussion

### Summary

This study provides a comprehensive analysis of factors influencing 2WW referrals and updates the evidence base. The strongest predictors of all-cancer 2WW referral rate included female sex , age ≥75 years, patients feeling involved in care decisions, and practice location in Northwest England (higher referral rates), and practices with a higher frequency of patients seeing a preferred GP (lower referral rates). Practices with higher proportions of people who currently smoke and Asian or Black ethnicity patients had fewer 2WW referrals for all cancers, with the strongest association observed for skin cancer, and for breast cancer (except for Black ethnic group). There were slightly more remote consultations compared with in-person appointments in 2021–2022, reflecting sustained COVID-19-related changes to primary care delivery.^32^ However, there was no association between remote consultations and 2WW referral rate.

### Strengths and limitations

This study used data from all English practices (excluding a small proportion with missing or atypical data), reducing sampling bias and increasing generalisability. It included the most comprehensive combination of patient and practice-related predictor variables compared with earlier studies.^8–15^ The use of MVR permitted adjustment for confounding factors, enabling more accurate interpretation of findings. For instance, people from ethnic minorities often reside in more socioeconomically deprived neighbourhoods,^23^ and accounting for this helps to discern whether deprivation or ethnic group is more influential on 2WW activity. Additionally, the sensitivity analyses provide a more nuanced understanding about factors that have an impact on 2WW referrals for four prevalent, high-mortality cancers.

Limitations include the use of practice-level data, which prevented the exploration of specific characteristics of patients receiving a 2WW referral and introducing the ecological fallacy.^33^ For example, higher odds of three or more GP consultations before 2WW referral have been reported for individuals with more comorbidities,^34,35^ but time to 2WW referral could not be assessed using practice-level data. Furthermore, the composite morbidity index precluded assessment of individual comorbidities on 2WW referrals in our analysis. This analysis also utilised data from the GPPS, which had a low response rate (32.2%), although data is considered representative of the general population.^36^ Finally, our results leave >50% of the variation in 2WW referrals unaccounted. Remaining variation may be random or unexplained,^37^ or attributed to the capacity and organisation of local secondary-care diagnostic services.^38^

### Comparison with existing literature

Cancer incidence increases with age,^8–10^ while individuals from Asian and Black ethnic backgrounds and those residing in more socioeconomically deprived communities have higher odds of stage III–IV diagnoses.^3,4^ Therefore, to improve equitable access to early diagnosis and advance survival outcomes, efforts are required to provide proportionately higher 2WW referral rates for these populations.

In this analysis, age ≥75 years and female sex predicted higher 2WW referrals, consistent with earlier studies.^8–12^ These findings align with expected age-related variation^8–10^ and evidence of higher cancer symptom awareness among women.^39,40^ Yet, age ≥75 years was associated with fewer breast 2WW referrals. Given that a quarter of invasive breast cancers occur after 75 years^41^, this indicates potential underutilisation of breast 2WW pathways for older women. It is possible that older women may perceive a lower cancer risk once their breast screening ends at age 70, potentially reducing primary care presentation rates.^41^

Moreover, we found lower 2WW referral rates for Asian and Black patients, particularly for skin and breast cancer, underlining persistent unmet healthcare needs. This finding is consistent with earlier research,^8,10^ where factors including lower symptom awareness and barriers to help-seeking (for example, language and embarrassment around speaking with a doctor) may contribute to this disparity.^3,42–44^

Round and colleagues ^11^ reported lower 2WW cancer detection in more socioeconomically deprived communities, while the present analysis found no association between deprivation and 2WW referral rates (except for lung cancer). A possible explanation is that our analysis controlled for smoking, a well-documented driver of socioeconomic inequality in cancer incidence^45^ that is linked with reduced help-seeking behaviours.^39,46,47^ Indeed, our analysis suggests no additional impact of deprivation on 2WW referral rates beyond the effects of smoking.

Another explanation for the discrepancy in 2WW referral outcomes by deprivation is the use of different primary outcomes. Round and colleagues utilised the 2WW cancer detection rate, which measures the percentage of cancers diagnosed from a 2WW referral (the sensitivity of selecting patients for urgent referral),^11^ whereas 2WW referral rate does not reflect subsequent cancer diagnoses. Round and colleagues’ finding of lower 2WW cancer detection with higher deprivation may represent higher multimorbidity rates (and subsequently, symptoms) in more deprived communities,^48,49^ precipitating 2WW referrals for non-cancerous conditions. Our analysis supports this rationale by showing that practices with a higher morbidity index had increased 2WW referral rates .

Conversely, other GP–patient interaction studies have reported lower urgent cancer referrals with increasing comorbidities,^10,50,51^ yet there is a paucity of English studies investigating this association. We found that higher deprivation predicted fewer lung 2WW referrals. Lung cancer is regarded as ‘difficult to diagnose’ because of overlapping symptoms with comorbidities (for example, chronic obstructive pulmonary disease), which are more prevalent within deprived communities^48^ and can predispose incidental cancer findings during routine scans.^52^ Previous research suggests that GPs familiar with their patients may misattribute symptoms to existing LTCs, reducing cancer suspicion.^14,15^ This corroborates our finding that frequency of seeing a preferred GP, a proxy measure for continuity of care, was strongly associated with fewer 2WW referrals.

Notably, practices with higher scores for patients feeling involved in treatment decisions, a proxy measure for shared decision making (SDM), had higher 2WW referral rates . Some evidence suggests that GPs utilising SDM by discussing the rationale, risks, and benefits of procedures have fewer diagnostic delays for lower-gastrointestinal cancer.^53^ Further, misalignment between GP and patients’ perceptions of symptoms can discourage re-presentation if symptoms persist.^54^ Hence, this novel finding suggest a potentially important but underexplored role for SDM in 2WW pathways.

Finally, an unexpected finding was that practices in Northwest England strongly predicted higher 2WW referral rates . North England has the highest cancer incidence because of its distribution of older age and deprived populations,^55^ thus GPs in Northwest England may have higher cancer suspicion. Furthermore, previous public cancer awareness campaigns^56,57^ have targeted Northwest England, which could also explain this finding.

### Implications for research and practice

Practices with higher 2WW referrals are associated with improved cancer survival, thus initiatives increasing 2WW referral rates are generally considered beneficial.^58^ Regional variation in 2WW referral rates may highlight learning from best-practice exemplars and inspire quality improvement initiatives to increase 2WW activity.^59^ Promisingly, we observed higher 2WW referral rates in postgraduate GP training practices, indicating that GP education can increase urgent referrals.

Moreover, public cancer awareness campaigns can increase symptomatic presentation to primary care.^60^ This analysis highlights priority populations for campaigns including individuals from Asian and Black ethnic backgrounds and people who smoke, who experience lower 2WW referral rates but higher odds of late-stage diagnoses. Utilising ‘community champions’ for culturally tailored messages^61^ and collaborating with smoking cessation and community pharmacy services may improve outreach to individuals reluctant to access primary care.^62^ Finally, breast cancer awareness campaigns should encourage women aged ≥70 years to remain vigilant for symptoms once their screening has ended.

However, NHS resource constraints necessitate responsible use of 2WW referrals to prevent system overwhelm.^58^ Hence, further research on 2WW detection rates (that is, the rate at which GPs select the ‘correct’ patients for 2WW referral), especially for patients with multimorbidity who have ambiguous symptoms, could improve referral efficiency (limiting over- or under-referral). Involving patients in diagnostic decisions through SDM or ‘patient decision aids’^63^ may enhance 2WW pathways. Specifically, SDM may assist GPs to more accurately identify cancer symptoms and reassure low-risk patients while encouraging timely re-presentation should symptoms persist.

To conclude, these findings and recommendations are salient and timely given the drive to reduce health inequalities exacerbated by COVID-19^64^ and the ambition to diagnose 75% of cancers in stage I–II by 2028.

## Data

The dataset relied on in this article is available from the corresponding author on reasonable request.
